# Moving things forward in Hodgkin lymphoma

**DOI:** 10.12688/f1000research.16077.1

**Published:** 2018-11-13

**Authors:** Paul J. Bröckelmann, Boris Böll

**Affiliations:** 1 German Hodgkin Study Group and Department I of Internal Medicine, University Hospital of Cologne, Cologne, 50937, Germany

**Keywords:** Hodgkin Lymphoma, chemotherapy, PET, checkpoint inhibition, PD1, PD1-L, CD30, brentuximab vedodin, nivolumab, pembrolizumab, relapsed patients, older patients

## Abstract

Arising from the immune system and located primarily in lymphoid organs, Hodgkin lymphoma (HL) is one of the most common cancers in young adults. Risk-adapted first-line treatment usually consisting of multi-agent chemotherapy and often incorporating consolidative radiation therapy aims at long-term cure. Although this is achieved in the vast majority of patients, therapy-related side effects such as organ damage, second cancers, and fatigue constitute considerable sequelae and outweigh HL as the cause of mortality after successful first-line treatment. In addition, intensive conventional therapy is seldom feasible in elderly or frail patients, diminishing chances of cure in this growing population of patients. The rapidly growing understanding of HL biology, innovative clinical trials, and the incorporation of novel drugs might help to overcome these obstacles in the management of HL. In this review, recent advances in the understanding and care of HL will be summarized with a focus on ongoing and future strategies which might help move things forward.

## Introduction

Classic Hodgkin lymphoma (HL) is a rather rare cancer of the lymphoid system and clinically presents with swollen lymph nodes or symptoms due to organ involvement of advanced-stage disease. It is one of the most common malignancies in young adults but occurs at all ages. Over the recent decades, a growing incidence in older people between 70 and 80 years of age in addition to the stable peak between 20 and 30 years of age was observed ^1^. In Western countries, HL occurs at an incidence of 2.5 new cases per 100,000 people per year, resulting in an expected 18,525 cases in Europe annually. Owing to high cure rates with risk-adapted first-line therapy, prevalence is high and an estimated 208,805 people were living with HL in the US in 2015 ^2^.

Depending on disease extent and presence of constitutional symptoms such as fevers, night sweats, or weight loss, HL is classified as early-stage favorable, early-stage unfavorable, and advanced-stage disease for the purpose of treatment allocation ^3^. Cure rates with multi-agent chemotherapy and often consolidative radiation therapy (RT) are high with long-term remission rates of 80% to 90%, depending on risk group, age, and treatment ^4– 7^. In patients with primary progressive or refractory (1 to 3% of cases, depending on treatment regimen) as well as relapsed disease, high-dose chemotherapy (HDCT) followed by autologous stem cell transplantation is administered if feasible and can result in long-term remission in up to 50% of cases ^8^. More recently, several targeted agents were investigated in this setting and the antibody-drug conjugate brentuximab vedotin (BV) and the checkpoint inhibitors nivolumab and pembrolizumab were approved for relapsed/refractory HL (rrHL) ^9– 11^.

Despite reductions of intensity of both chemotherapy and RT in recent years, therapy-related short- and long-term morbidity constitutes considerable sequelae and these side effects surmount HL mortality over the years ^12^. Besides organ damage such as pulmonary or cardiovascular disease as well as infertility, second cancers and long-term fatigue are of particular clinical relevance to patients and caregivers ^13– 15^. To minimize these complications, a major goal of current research is to further refine first-line treatment to develop equally effective but less intensive strategies. Ideally, those therapeutic approaches would also be feasible for the growing population of older or frail patients for whom prognosis is less favorable ^16, 17^.

The present review summarizes recent advances in understanding and the current approach to managing HL. We also address remaining challenges and outline ongoing as well as future efforts to move things forward in HL over the next years.

## Hodgkin lymphoma biology

Characterized by a paucity of the malignant Hodgkin and Reed–Sternberg (HRS) cells in an abundant (micro)environment of immune cells, HL is very distinct from most other cancers and lymphomas ^18^. The B-cell precursor heritage of HRS cells was long acknowledged, whereas a critical influence of immune checkpoint inhibition via the programmed death 1 (PD1) and PD1-ligand (PD-L1) was only recently reported ^19^. Owing to amplification or copy gain of 9p24.1 ^20^ or mediated by Epstein–Barr virus (EBV) infection ^21^, HRS cells frequently express PD-L1 and thereby evade a sustained anti-tumor response of the patients’ immune system. In addition, HRS cells frequently lack major histocompatibility class I (MHC I) expression because of mutations of beta-2-microglobulin (β2M) ^22^. The HL microenvironment is characterized predominantly by T and natural killer cells as well as macrophages, and PD-1 expression occurs in the former ^23^. The latter are often PD-L1 ^+^ and associated with inferior outcomes with conventional therapies ^24, 25^. A very recent characterization of tumor-associated T cells via a customized time-of-flight mass cytometry (CyTOF) revealed the presence of PD-1 ^+^ CD4 ^+^ effector and PD-1 ^−^ CD4 ^+^ regulatory T cells as potential complementary mechanisms of local immunosuppression ^26^. This rapidly growing understanding of the tumor composition is complemented by the possibility to identify and track cell-free tumor DNA in the blood ^27^. Although this technique potentially allows non-invasive monitoring of disease activity, it also constitutes the possibility to evaluate the genetic background of HL, which earlier was compromised by the low tumor cell content in biopsies. In summary, recent studies were able to shed light on the distinct biology underlying HL which will help to further develop targeted therapies and synergistic therapeutic concepts.

## Recent developments in first-line treatment

### Early-stage disease

Patients with limited stage I–II disease (that is, involvement of lymph nodes on only one side of the diaphragm and without extra-lymphatic disease) are grouped into early-stage favorable and unfavorable HL for the purpose of first-line therapy. Whereas those with clinical risk factors (RFs) such as involvement of at least three lymphatic areas, an elevated erythrocyte sedimentation rate, extra-nodal (EN) involvement, or a large mediastinal mass (LMM) are considered unfavorable risk, the remaining patients are usually considered a favorable risk group. In contrast to disseminated extra-lymphatic disease, which defines stage IV disease, EN involvement is defined as local limited contiguous tumor spread from a lymphatic HL manifestation. Despite nuances in the RFs used to determine early-stage unfavorable disease, the three major currently applied classifications retain their prognostic impact with conventional treatment ^28^.

Already two decades ago, combined modality treatment consisting of two to four cycles of, in most cases, doxorubicin, bleomycin, vinblastine, dacarbazine (ABVD) chemotherapy followed by consolidative RT was established for both early-stage risk groups ^29, 30^. Consecutively, the initially large RT fields could be markedly reduced from subtotal nodal irradiation (STNI) to involved-field (IF-RT) and lately involved-site or -node (IS-/IN-RT) without any loss in efficacy ^31, 32^. Omission of consolidative RT irrespective of response to chemotherapy results in an impaired disease control, and a recent registry-based analysis reported inferior overall survival (OS) ^33, 34^. Therefore, several randomized trials evaluate the omission of consolidative RT in patients who attain a complete metabolic response— ^18^F-FDG positron emission tomography (PET) negativity—with systemic therapy.

In the UK RAPID trial, PET-negative patients after 3xABVD who did not receive consolidative 30 Gy IF-RT due to randomization into the experimental arm had inferior three-year progression-free survival (PFS) with a three-year OS similar to that of those in the standard arm (per-protocol analysis: 97.1% versus 90.8%) ^35^. A similar observation was made in the EORTC H10F/U trial. In the experimental arms, patients with early-stage favorable or unfavorable disease received two or four additional cycles of ABVD if PET-negative after 2xABVD instead of one or two additional cycles of ABVD and consolidative 30 Gy IN-RT, respectively. Inferior five-year PFSs of 99.0% versus 87.1% and 92.1% versus 89.6% for patients with favorable and unfavorable early-stage disease (respectively) were reported, whereas until now no differences in OS were reported ^36^.

The two large randomized phase III trials—HD16 (early-stage favorable) and HD17 (early-stage unfavorable)—evaluating non-inferiority of PET-adapted omission of consolidative RT recently completed recruitment. Of note, PET status is evaluated after completion of systemic therapy with either 2xABVD or 2xABVD + 2xBEACOPP _escalated_ in both trials and no further therapy administered in PET-negative patients in contrast to the RAPID or H10 trials. Results are eagerly awaited to inform treating physicians and patients since, to date, omission of RT cannot generally be recommended if optimal disease control is the main goal of therapy. Since differences in three- and five-year PFS are rather small, especially in patients with early-stage unfavorable disease, omission of RT may be considered in individual cases after risks and benefits are weighed. This could apply, for example, to a young female patient with axillary lymph nodes prompting relevant RT volume and dose to healthy breast tissue or to a young man with a family history of cardiac disease requiring relevant RT exposure to his coronary arteries because of lower mediastinal HL involvement.

For patients with early-stage favorable HL, a recent long-term analysis confirmed the previously reported favorable outcome with only 2xABVD + 20 Gy consolidative RT ^7^. In patients with early-stage unfavorable HL, outcome with ABVD-based systemic therapy is less favorable with 10-year PFS of only 84% with 4xABVD + 30 Gy IF-RT, underlining the need for effective therapies in this patient population. Lately, the randomized HD14 trial established a combination of 2xBEACOPP _escalated_ + 2xABVD (“2+2”) as a highly effective approach with five-year PFS of 95.4% in comparison with the standard of 4xABVD (five-year PFS of 89.1%) ^5^. More recently, the abovementioned H10U trial showed promising five-year PFS of 90.6% with an intensification of therapy by 2xBEACOPP _escalated_ for the unfavorable subgroup of interim PET-positive patients after initial 2xABVD ^36^.

### Advanced-stage disease

Patients with involvement of lymph nodes on both sides of the diaphragm, disseminated organ, or bone marrow involvement are considered to have advanced-stage disease by all classification systems. Some groups additionally consider patients with stage I–II disease and a combination of constitutional B symptoms and the RFs of LMM or EN disease (or both) as having advanced-stage disease. Initial treatment in this risk group is guided by an interim PET after two cycles of systemic therapy (PET-2) and consists of up to eight cycles of multi-agent chemotherapy and localized RT to PET-positive residues thereafter.

Choice of systemic therapy is a matter of long-lasting and ongoing debate: ABVD is associated with considerably lower acute and long-term toxicity and, in contrast to BEACOPP _escalated_, potentially suitable for patients older than 60 years of age. Long-term toxicities more frequently occurring after BEACOPP-based therapy are sterility and occurrence of second cancers. However, the risks to develop refractory disease or relapse of HL are significantly higher with ABVD, and a recent network meta-analysis confirmed an OS benefit for patients treated with the more intensive regimen ^37^. To develop individualized approaches, the three recently reported large randomized phase III RATHL, HD18 and AHL2011 trials investigated initial therapy with ABVD or BEACOPP _escalated_ guided by interim PET.

In the experimental arm of the British RATHL, PET-2–negative patients received +4xAVD to avoid bleomycin-associated pulmonary toxicity, and in PET-2–positive patients, therapy was intensified by 4xBEACOPP ^38^. Whereas the de-escalation to AVD proved to be non-inferior in terms of three-year PFS, intensification resulted in three-year PFS of 67.5%. Importantly, escalation was not subject to randomization and only historical comparisons suggest that intensification with BEACOPP _escalated_ might be superior to continuation with ABVD in PET-2–positive patients. In the GHSG HD18, a de-escalation strategy with patients PET-2–negative after 2xBEACOPP _escalated_ randomly assigned to receive either +4x or +6–8xBEACOPP _escalated_ was investigated. The experimental strategy was not inferior with five-year PFS of 92.2% versus 90.8% and five-year OS of 97.7% versus 95.4% for the standard approach ^6^. The French AHL2011 trial recently reported a non-inferior four-year PFS of 87.1% with randomized de-escalation to 4xABVD in patients who achieved a PET-2–negative status after 2xBEACOPP _escalated_ versus 87.4% with full 6xBEACOPP _escalated_^39^.

Conclusions from across trial comparisons are limited by differences in risk-group definitions, patient populations, and the varying clinical setting and hence are impossible. Nevertheless, it may be concluded from the recent data that PET-2 is an important reliable tool to guide therapy in advanced-stage HL, and one might get the impression that an initial therapy with BEACOPP _escalated_ results in long-term outcomes superior to those of ABVD-based strategies.

## Drugs beyond conventional chemotherapy

Over many decades, no new drugs were approved for the treatment of HL. That changed in 2011, when the anti-CD30 antibody drug conjugate BV was approved for the treatment of rrHL on the basis of a pivotal phase II trial showing an overall response rate (ORR) of 75% and complete response rate (CRR) of 34% ^10^. Recent follow-up analyses revealed sustained remissions in patients achieving a CR with five-year PFS of 52%. Interestingly, 9% of all patients maintained a CR even without consolidative stem cell transplantation (SCT) ^40^.

To improve efficacy of ABVD in patients with advanced-stage HL, a combination of BV and AVD was investigated in the company-sponsored randomized phase III ECHELON-1 trial: With a superior two-year modified PFS of 82.1% versus 77.1%, 6x BV-AVD was considered superior to 6xABVD, but more mature follow-up or conventional PFS data have not yet been reported and concerns have been expressed over the risk-to-benefit ratio of BV-AVD given the higher toxicity of the novel regimen ^41, 42^. In patients with early-stage HL, consolidative therapy with BV instead of RT after systemic therapy with ABVD was investigated in a phase II trial and associated with one-year PFS and OS rates of 91% and 95%, respectively, in an early analysis ^43^. A sequential approach with BV prior to and after AVD in elderly patients with treatment-naïve HL was tolerable and resulted in two-year PFS and OS rates of 84% and 93%, respectively ^44^.

More recently, the two checkpoint inhibitors nivolumab and pembrolizumab targeting PD1 on exhausted T and other immune cells were approved for rrHL. Nivolumab and pembrolizumab showed ORRs of 69% and 65%, respectively, and a CRR of 16% with long-lasting responses in patients achieving a partial remission (PR) in the pivotal phase II trials ^9, 11^. Correlative studies revealed the prognostic relevance of PD-L1 and MHC II expression by HRS cells, shedding light on the mechanisms involved in responses in a heavily pretreated patient population ^45^. While increased PD-L1 expression was associated with improved PFS, patients with less PD-L1 expression benefited from nivolumab and hence checkpoint inhibition is administered irrespective of the expression profile. Early data of an ongoing trial investigating the combination of nivolumab with BV in rrHL show an ORR of 85% and CRR of 65% while, despite manageable infusion-related reactions, tolerability was good ^46^. In addition, the first results without relevant follow-up were recently reported for the combination of nivolumab and AVD in advanced-stage HL: Of 51 patients, 90% were able to complete the planned treatment with 4x nivolumab followed by 6x Nivo-AVD, and the ORR was 84% with a CRR of 67% by independent review committee ^47^.

In light of the changing therapeutic landscape, other agents such as bendamustine or lenalidomide experience a revival and—since they proved to be considerably active in rrHL— are increasingly used in clinical practice ^48, 49^. In addition, drugs originally approved for other hematological cancers, such as the histone deacetylase inhibitors panobinstat or mocetinostat or the inhibitor of PI3Kδ idelalisib, have been investigated in rrHL with relevant efficacy and manageable toxicity ^50– 52^. A welcomed side effect of the growing armory of effective drugs is the possibility to conduct both autologous and allogeneic SCT increasingly in patients with at least PR, which is associated with a more favorable prognosis ^3^. Initial reports raised concerns due to toxicity and mortality associated with high-grade graft-versus-host disease associated with allogeneic stem cell transplantation (alloSCT) after reinduction therapy with an anti-PD1 ^53^. More recent data indicate that alloSCT is feasible and associated with high CRR and prolonged remissions. The previously postulated prognostic impact of time between last dose of the anti-PD1 antibody could not be confirmed, and data from larger series are still unavailable ^54^.

Chimeric antigen receptor (CAR) T cells were recently approved for the treatment of other hematologic cancers such as acute lymphoblastic leukemia or B-cell non-HL. Two early-phase trials very recently reported manageable toxicities after infusion of autologous anti-CD30 CAR T cells in patients with rrHL. Investigators from Houston reported two patients with sustained CR (CRR of 29%) and stabilization of disease (SD) in another two of the seven patients treated ^55^. In a Chinese trial, PR was observed in seven and SD in six among 18 patients treated without any CRs documented yet ^56^. Aimed at the EBV antigen latent membrane proteins, another CAR construct from Houston was tolerable and effective as adjuvant or re-induction therapy in patients with EBV-associated lymphoma ^57^. Another construct is targeting CD19 in the HL tumor microenvironment and on circulating CD19-positive HRS precursor cells and among four patients treated, one CR and one PR with consecutive progressive disease were observed ^58^. Promising preclinical activity was additionally reported for CARs targeting CD123 to affect both the HRS cells and microenvironment ^59^.

## Selected ongoing first-line trials

While numerous phase II trials investigate various combinations of novel agents with conventional chemotherapy or each other in rrHL, the trial landscape is scarcer in the first-line setting. Investigating innovative therapies for these patients is challenging since current approaches bear mostly excellent long-term outcome. Decreasing intensity within the controlled setting of prospective trials previously resulted in significantly inferior disease control. However, an impaired OS was not reported and this was likely due to effective salvage therapies and no increase in late reoccurrences of HL observed so far ^4, 31, 60^.

To improve results in patients with early-stage unfavorable HL without exposing them to the toxicity of “2+2”, two ongoing investigator-initiated phase II trials investigate innovative strategies. In the randomized GHSG NIVAHL trial, patients receive the combination of nivolumab and AVD as either 4xNivo-AVD or 4x nivolumab + 2xNivo-AVD + 2xAVD, each followed by consolidative 30 Gy IS-RT (ClinicalTrials.gov Identifier: NCT03004833). A combination of AVD with BV is investigated in a US-based trial with a focus on consolidative RT (ClinicalTrials.gov Identifier: NCT01868451). Assigned to one of four different cohorts, patients will receive 30 Gy IS-RT, 20 Gy IS-RT, 30.6 Gy consolidation volume RT, or no RT if PET negativity is achieved after 4xBV-AVD.

In advanced-stage disease, the multinational randomized phase III trial HD21 is comparing a modified BEACOPP-regimen incorporating BV, dacarbazine, and dexamethasone instead of bleomycin, vincristine, procarbazine, and prednisolone (BRECADD) ^61^ versus BEACOPP _escalated_. The trial is aiming to show non-inferiority in terms of efficacy and superiority in terms of treatment-related morbidity (ClinicalTrials.gov Identifier: NCT02661503), thereby better balancing risk and benefits of an intensified first-line treatment. As the CHOP (cyclophosphamide, doxorubicin, prednisone, and vincristine) regimen is well tolerated in elderly patients with non-HL ^62^, the combination of BV with a CHOP-like regimen is being investigated in the phase-II B-CAP trial for elderly patients with advanced-stage HL (ClinicalTrials.gov Identifier: NCT02191930).

## Future perspectives

First-line treatment of HL with multi-agent chemotherapy and often consolidative RT is well established and results in cure in the majority of patients eligible for intensive therapies. Nevertheless, prognosis is less favorable in frail or older patients, and long-term side effects such as second cancers, organ damage, or fatigue as well as rrHL constitute considerable sequelae. The approval of modern drugs such as BV, nivolumab, and pembrolizumab for rrHL allow ongoing and future research to develop at least equally effective but less toxic therapies. Increasing understanding of PET imaging also as a potential prognostic marker by assessment of the metabolic tumor volume allows increased patient stratification and individualization of the therapeutic sequence. A growing understanding of the underlying biology, as well as the possibility to evaluate genetic alterations and monitor activity of disease by cell-free tumor DNA, additionally helps in doing so. From this point forward, it is crucial to keep in mind the currently achieved outcomes with conventional approaches. Carefully refining HL therapy meeting the needs and primary goals of affected patients is challenging, and ideally changes in clinical routine should be informed by evidence from randomized phase III trials.

## Abbreviations

ABVD, doxorubicin, bleomycin, vinblastine, dacarbazine; alloSCT, allogeneic stem cell transplantation; AVD, doxorubicin, vinblastine, dacarbazine; BEACOPP _escalated_, bleomycin, etoposide, doxorubicin, cyclophosphamide, vinblastine, procarbazine, prednisone; BRECADD, brentuximab vedotin, etoposide, cyclophosphamide, doxorubicin, dacarbazine, dexamethasone; BV, brentuximab vedotin; CAR, chimeric antigen receptor; CHOP, cyclophosphamide, doxorubicin, prednisone, and vincristine; CR, complete response; CRR, complete response rate; EBV, Epstein–Barr virus; EN, extra-nodal; HL, Hodgkin lymphoma; HRS, Hodgkin and Reed–Sternberg; IF-RT, involved-field radiation therapy; IN-RT, involved-node radiation therapy; IS-RT, involved-site radiation therapy; LMM, large mediastinal mass; MHC, major histocompatibility class; Nivo, nivolumab; ORR, overall response rate; OS, overall survival; PD-1, programmed-death receptor 1; PD-L1, programmed-death receptor 1 ligand; PET, ^18^F-FDG positron emission tomography; PFS, progression-free survival; PR, partial remission; RF, risk factor; rrHL, relapsed or refractory Hodgkin lymphoma; RT, radiation therapy; SCT, stem cell transplantation; SD, stabilization of disease

## References

[1] Robert Koch Institut: Krebs in Deutschland, ICD-10 C81 Morbus Hodgkin.2013;112–115. Reference Source

[2] Siegel RL Miller KD Jemal A: Cancer statistics, 2015. *CA Cancer J Clin.* 2015;65(1):5–29. 10.3322/caac.21254 25559415

[3] Bröckelmann PJ Angelopoulou MK Vassilakopoulos TP: Prognostic factors in Hodgkin lymphoma. *Semin Hematol.* 2016;53(3):155–64. 10.1053/j.seminhematol.2016.05.003 27496306

[4] Behringer K Goergen H Hitz F: Omission of dacarbazine or bleomycin, or both, from the ABVD regimen in treatment of early-stage favourable Hodgkin's lymphoma (GHSG HD13): an open-label, randomised, non-inferiority trial. *Lancet.* 2015;385(9976):1418–27. 10.1016/S0140-6736(14)61469-0 25539730

[5] von Tresckow B Plütschow A Fuchs M: Dose-intensification in early unfavorable Hodgkin's lymphoma: final analysis of the German Hodgkin Study Group HD14 trial. *J Clin Oncol.* 2012;30(9):907–13. 10.1200/JCO.2011.38.5807 22271480

[6] Borchmann P Goergen H Kobe C: PET-guided treatment in patients with advanced-stage Hodgkin's lymphoma (HD18): final results of an open-label, international, randomised phase 3 trial by the German Hodgkin Study Group. *Lancet.* 2018;390(10114):2790–802. 10.1016/S0140-6736(17)32134-7 29061295

[7] Sasse S Bröckelmann PJ Goergen H: Long-Term Follow-Up of Contemporary Treatment in Early-Stage Hodgkin Lymphoma: Updated Analyses of the German Hodgkin Study Group HD7, HD8, HD10, and HD11 Trials. *J Clin Oncol.* 2017;35(18):1999–2007. 10.1200/JCO.2016.70.9410 28418763

[8] Josting A Müller H Borchmann P: Dose intensity of chemotherapy in patients with relapsed Hodgkin's lymphoma. *J Clin Oncol.* 2010;28(34):5074–80. 10.1200/JCO.2010.30.5771 20975066

[9] Younes A Santoro A Shipp M: Nivolumab for classical Hodgkin's lymphoma after failure of both autologous stem-cell transplantation and brentuximab vedotin: a multicentre, multicohort, single-arm phase 2 trial. *Lancet Oncol.* 2016;17(9):1283–94. 10.1016/S1470-2045(16)30167-X 27451390 PMC5541855

[10] Younes A Gopal AK Smith SE: Results of a pivotal phase II study of brentuximab vedotin for patients with relapsed or refractory Hodgkin's lymphoma. *J Clin Oncol.* 2012;30(18):2183–9. 10.1200/JCO.2011.38.0410 22454421 PMC3646316

[11] Chen R Zinzani PL Fanale MA: Phase II Study of the Efficacy and Safety of Pembrolizumab for Relapsed/Refractory Classic Hodgkin Lymphoma. *J Clin Oncol.* 2017;35(19):2125–32. 10.1200/JCO.2016.72.1316 28441111 PMC5791843

[12] Ng AK: Current survivorship recommendations for patients with Hodgkin lymphoma: focus on late effects. *Blood.* 2014;124(23):3373–9. 10.1182/blood-2014-05-579193 25428219

[13] Schaapveld M Aleman BM van Eggermond AM: Second Cancer Risk Up to 40 Years after Treatment for Hodgkin's Lymphoma. *N Engl J Med.* 2015;373(26):2499–511. 10.1056/NEJMoa1505949 26699166

[14] Kreissl S Mueller H Goergen H: Cancer-related fatigue in patients with and survivors of Hodgkin's lymphoma: a longitudinal study of the German Hodgkin Study Group. *Lancet Oncol.* 2016;17(10):1453–62. 10.1016/S1470-2045(16)30093-6 27612583

[15] Bhakta N Liu Q Yeo F: Cumulative burden of cardiovascular morbidity in paediatric, adolescent, and young adult survivors of Hodgkin's lymphoma: an analysis from the St Jude Lifetime Cohort Study. *Lancet Oncol.* 2016;17(9):1325–34. 10.1016/S1470-2045(16)30215-7 27470081 PMC5029267

[16] Borchmann S Engert A Böll B: Hodgkin lymphoma in elderly patients. *Curr Opin Oncol.* 2018;30(5):308–16. 10.1097/CCO.0000000000000464 29994901

[17] Böll B Görgen H Fuchs M: ABVD in older patients with early-stage Hodgkin lymphoma treated within the German Hodgkin Study Group HD10 and HD11 trials. *J Clin Oncol.* 2013;31(12):1522–9. 10.1200/JCO.2012.45.4181 23509310

[18] Swerdlow SH Campo E Pileri SA: The 2016 revision of the World Health Organization classification of lymphoid neoplasms. *Blood.* 2016;127(20):2375–90. 10.1182/blood-2016-01-643569 26980727 PMC4874220

[19] Yamamoto R Nishikori M Kitawaki T: PD-1-PD-1 ligand interaction contributes to immunosuppressive microenvironment of Hodgkin lymphoma. *Blood.* 2008;111(6):3220–4. 10.1182/blood-2007-05-085159 18203952

[20] Roemer MG Advani RH Ligon AH: *PD-L1* and *PD-L2* Genetic Alterations Define Classical Hodgkin Lymphoma and Predict Outcome. *J Clin Oncol.* 2016;34(23):2690–7. 10.1200/JCO.2016.66.4482 27069084 PMC5019753

[21] Green MR Rodig S Juszczynski P: Constitutive AP-1 activity and EBV infection induce PD-L1 in Hodgkin lymphomas and posttransplant lymphoproliferative disorders: implications for targeted therapy. *Clin Cancer Res.* 2012;18(6):1611–8. 10.1158/1078-0432.CCR-11-1942 22271878 PMC3321508

[22] Reichel J Chadburn A Rubinstein PG: Flow sorting and exome sequencing reveal the oncogenome of primary Hodgkin and Reed-Sternberg cells. *Blood.* 2015;125(7):1061–72. 10.1182/blood-2014-11-610436 25488972

[23] Muenst S Hoeller S Dirnhofer S: Increased programmed death-1+ tumor-infiltrating lymphocytes in classical Hodgkin lymphoma substantiate reduced overall survival. *Hum Pathol.* 2009;40(12):1715–22. 10.1016/j.humpath.2009.03.025 19695683

[24] Vari F Arpon D Keane C: Immune evasion via PD-1/PD-L1 on NK cells and monocyte/macrophages is more prominent in Hodgkin lymphoma than DLBCL. *Blood.* 2018;131(16):1809–19. 10.1182/blood-2017-07-796342 29449276 PMC5922274

[25] Steidl C Lee T Shah SP: Tumor-associated macrophages and survival in classic Hodgkin's lymphoma. *N Engl J Med.* 2010;362(10):875–85. 10.1056/NEJMoa0905680 20220182 PMC2897174

[26] Cader FZ Schackmann RCJ Hu X: Mass cytometry of Hodgkin lymphoma reveals a CD4 ^+^ regulatory T-cell-rich and exhausted T-effector microenvironment. *Blood.* 2018;132(8):825–36. 10.1182/blood-2018-04-843714 29880615 PMC6107878

[27] Vandenberghe P Wlodarska I Tousseyn T: Non-invasive detection of genomic imbalances in Hodgkin/Reed-Sternberg cells in early and advanced stage Hodgkin's lymphoma by sequencing of circulating cell-free DNA: A technical proof-of-principle study. *Lancet Haematol.* 2015;2(2):e55–65. 10.1016/S2352-3026(14)00039-8 26687610

[28] Klimm B Goergen H Fuchs M: Impact of risk factors on outcomes in early-stage Hodgkin's lymphoma: an analysis of international staging definitions. *Ann Oncol.* 2013;24(12):3070–6. 10.1093/annonc/mdt413 24148816

[29] Engert A Franklin J Eich HT: Two cycles of doxorubicin, bleomycin, vinblastine, and dacarbazine plus extended-field radiotherapy is superior to radiotherapy alone in early favorable Hodgkin's lymphoma: final results of the GHSG HD7 trial. *J Clin Oncol.* 2007;25(23):3495–502. 10.1200/JCO.2006.07.0482 17606976

[30] Noordijk EM Carde P Dupouy N: Combined-modality therapy for clinical stage I or II Hodgkin's lymphoma: long-term results of the European Organisation for Research and Treatment of Cancer H7 randomized controlled trials. *J Clin Oncol.* 2006;24(19):3128–35. 10.1200/JCO.2005.05.2746 16754934

[31] Engert A Plütschow A Eich HT: Reduced treatment intensity in patients with early-stage Hodgkin's lymphoma. *N Engl J Med.* 2010;363(7):640–52. 10.1056/NEJMoa1000067 20818855

[32] Specht L Yahalom J Illidge T: Modern radiation therapy for Hodgkin lymphoma: field and dose guidelines from the international lymphoma radiation oncology group (ILROG). *Int J Radiat Oncol Biol Phys.* 2014;89(4):854–62. 10.1016/j.ijrobp.2013.05.005 23790512

[33] Hay AE Klimm B Chen BE: An individual patient-data comparison of combined modality therapy and ABVD alone for patients with limited-stage Hodgkin lymphoma. *Ann Oncol.* 2013;24(12):3065–9. 10.1093/annonc/mdt389 24121121

[34] Olszewski AJ Shrestha R Castillo JJ: Treatment selection and outcomes in early-stage classical Hodgkin lymphoma: analysis of the National Cancer Data Base. *J Clin Oncol.* 2015;33(6):625–33. 10.1200/JCO.2014.58.7543 25584010

[35] Radford J Barrington S Counsell N: Involved Field Radiotherapy Versus No Further Treatment in Patients with Clinical Stages IA and IIA Hodgkin Lymphoma and a 'Negative' PET Scan After 3 Cycles ABVD. Results of the UK NCRI RAPID Trial.ASH Annual Meeting Abstracts.2012;120:547 Reference Source

[36] André MPE Girinsky T Federico M: Early Positron Emission Tomography Response-Adapted Treatment in Stage I and II Hodgkin Lymphoma: Final Results of the Randomized EORTC/LYSA/FIL H10 Trial. *J Clin Oncol.* 2017;35(16):1786–94. 10.1200/JCO.2016.68.6394 28291393

[37] Skoetz N Trelle S Rancea M: Effect of initial treatment strategy on survival of patients with advanced-stage Hodgkin's lymphoma: a systematic review and network meta-analysis. *Lancet Oncol.* 2013;14(10):943–52. 10.1016/S1470-2045(13)70341-3 23948348

[38] Johnson P Federico M Kirkwood A: Adapted Treatment Guided by Interim PET-CT Scan in Advanced Hodgkin's Lymphoma. *N Engl J Med.* 2016;374(25):2419–29. 10.1056/NEJMoa1510093 27332902 PMC4961236

[39] Casasnovas O Brice P Bouabdallah R: FINAL ANALYSIS OF THE AHL2011 RANDOMIZED PHASE III LYSA STUDY COMPARING AN EARLY PET DRIVEN TREATMENT DE-ESCALATION TO A NOT PET-MONITORED STRATEGY IN PATIENTS WITH ADVANCED STAGES HODGKIN LYMPHOMA. *EHA Learning Center.* 2018;214504 Reference Source

[40] Chen R Gopal AK Smith SE: Five-year survival and durability results of brentuximab vedotin in patients with relapsed or refractory Hodgkin lymphoma. *Blood.* 2016;128(12):1562–6. 10.1182/blood-2016-02-699850 27432875 PMC5034737

[41] Borchmann P Fosså A Długosz-Danecka M: The Phase 3 Study ECHELON-1 Evaluating Brentuximab Vedotin in Patients With Newly Diagnosed Hodgkin Lymphoma Leaves Important Questions Unanswered. *HemaSphere.* 2018;2(3):e52 10.1097/HS9.0000000000000052 PMC6745994 31723777

[42] Adams HJA Kwee TC: Benefit of brentuximab over bleomycin in first-line treatment of advanced-stage Hodgkin lymphoma has not been proven. *Blood.* 2018;132(3):339–40. 10.1182/blood-2018-04-845438 29858234

[43] Park SI Olajide OA Reddy NM: A phase 2 trial of ABVD followed by brentuximab vedotin consolidation in limited stage non-bulky Hodgkin lymphoma. *JCO.* 2016;34(15_suppl):7508 10.1200/JCO.2016.34.15_suppl.7508

[44] Evens AM Advani RH Helenowski IB: Multicenter Phase II Study of Sequential Brentuximab Vedotin and Doxorubicin, Vinblastine, and Dacarbazine Chemotherapy for Older Patients With Untreated Classical Hodgkin Lymphoma. *J Clin Oncol.* 2018;36(30):JCO2018790139. 10.1200/JCO.2018.79.0139 30179569

[45] Roemer MGM Redd RA Cader FZ: Major Histocompatibility Complex Class II and Programmed Death Ligand 1 Expression Predict Outcome After Programmed Death 1 Blockade in Classic Hodgkin Lymphoma. *J Clin Oncol.* 2018;36(10):942–50. 10.1200/JCO.2017.77.3994 29394125 PMC5877802

[46] Herrera AF Moskowitz AJ Bartlett NL: Interim results of brentuximab vedotin in combination with nivolumab in patients with relapsed or refractory Hodgkin lymphoma. *Blood.* 2018;131(11):1183–94. 10.1182/blood-2017-10-811224 29229594 PMC5855021

[47] Ramchandren R Domenech ED Rueda A: CHECKMATE 205 COHORT D: A PHASE 2 TRIAL OF NIVOLUMAB FOR NEWLY DIAGNOSED ADVANCED-STAGE CLASSICAL HODGKIN LYMPHOMA.EHA Learning Center.2018;214507 Reference Source

[48] Moskowitz AJ Hamlin PA Jr Perales MA: Phase II study of bendamustine in relapsed and refractory Hodgkin lymphoma. *J Clin Oncol.* 2013;31(4):456–60. 10.1200/JCO.2012.45.3308 23248254 PMC3862960

[49] Böll B Borchmann P Topp MS: Lenalidomide in patients with refractory or multiple relapsed Hodgkin lymphoma. *Br J Haematol.* 2010;148(3):480–2. 10.1111/j.1365-2141.2009.07963.x 19863533

[50] Younes A Sureda A Ben-Yehuda D: Panobinostat in patients with relapsed/refractory Hodgkin's lymphoma after autologous stem-cell transplantation: results of a phase II study. *J Clin Oncol.* 2012;30(18):2197–203. 10.1200/JCO.2011.38.1350 22547596

[51] Gopal AK Fanale MA Moskowitz CH: Phase II study of idelalisib, a selective inhibitor of PI3Kδ, for relapsed/refractory classical Hodgkin lymphoma. *Ann Oncol.* 2017;28(5):1057–63. 10.1093/annonc/mdx028 28327905 PMC6246229

[52] Younes A Oki Y Bociek RG: Mocetinostat for relapsed classical Hodgkin's lymphoma: an open-label, single-arm, phase 2 trial. *Lancet Oncol.* 2011;12(13):1222–8. 10.1016/S1470-2045(11)70265-0 22033282 PMC5042214

[53] Armand P Zinzani PL Collins GP: Outcomes of Allogeneic Hematopoietic Stem Cell Transplantation (HSCT) after Treatment with Nivolumab for Relapsed/Refractory Hodgkin Lymphoma. *Blood.* 2016;128:3502 Reference Source

[54] Mear J-B Nicolas-Virelizier E Schiano JM: ALLOGENEIC HEMATOPOIETIC STEM CELL TRANSPLANTATION AFTER NIVOLUMAB TREATMENT IN RELAPSED/REFRACTORY HODGKIN LYMPHOMA.EHA Learning Center.2018;214889 Reference Source

[55] Ramos CA Ballard B Zhang H: Clinical and immunological responses after CD30-specific chimeric antigen receptor-redirected lymphocytes. *J Clin Invest.* 2017;127(9):3462–71. 10.1172/JCI94306 28805662 PMC5669573

[56] Wang CM Wu ZQ Wang Y: Autologous T Cells Expressing CD30 Chimeric Antigen Receptors for Relapsed or Refractory Hodgkin Lymphoma: An Open-Label Phase I Trial. *Clin Cancer Res.* 2017;23(5):1156–66. 10.1158/1078-0432.CCR-16-1365 27582488

[57] Bollard CM Gottschalk S Torrano V: Sustained complete responses in patients with lymphoma receiving autologous cytotoxic T lymphocytes targeting Epstein-Barr virus latent membrane proteins. *J Clin Oncol.* 2014;32(8):798–808. 10.1200/JCO.2013.51.5304 24344220 PMC3940538

[58] Svoboda J Rheingold SR Gill SI: Pilot Study of Non-Viral, RNA-Redirected Autologous Anti-CD19 Chimeric Antigen Receptor Modified T-Cells in Patients with Refractory/Relapsed Hodgkin Lymphoma (HL). *Blood.* 2017;130:653–653.

[59] Ruella M Klichinsky M Kenderian SS: Overcoming the Immunosuppressive Tumor Microenvironment of Hodgkin Lymphoma Using Chimeric Antigen Receptor T Cells. *Cancer Discov.* 2017;7(10):1154–67. 10.1158/2159-8290.CD-16-0850 28576927 PMC5628114

[60] Bröckelmann PJ Goergen H Kohnhorst C: Late Relapse of Classical Hodgkin Lymphoma: An Analysis of the German Hodgkin Study Group HD7 to HD12 Trials. *J Clin Oncol.* 2017;35(13):1444–50. 10.1200/JCO.2016.71.3289 28240973

[61] Eichenauer DA Plütschow A Kreissl S: Incorporation of brentuximab vedotin into first-line treatment of advanced classical Hodgkin's lymphoma: final analysis of a phase 2 randomised trial by the German Hodgkin Study Group. *Lancet Oncol.* 2017;18(12):1680–7. 10.1016/S1470-2045(17)30696-4 29133014

[62] Kolstad A Nome O Delabie J: Standard CHOP-21 as first line therapy for elderly patients with Hodgkin's lymphoma. *Leuk Lymphoma.* 2007;48(3):570–6. 10.1080/10428190601126610 17454601

